# Special Issue “Biopolymers in Drug and Gene Delivery Systems 3.0”

**DOI:** 10.3390/ijms26189238

**Published:** 2025-09-22

**Authors:** Yury A. Skorik

**Affiliations:** Branch of Petersburg Nuclear Physics Institute named by B.P. Konstantinov of National Research Centre “Kurchatov Institute”—Institute of Macromolecular Compounds, Bolshoi VO 31, St. Petersburg 199004, Russia; yury_skorik@mail.ru

The evolution of biopolymer-based drug and gene delivery systems is a compelling narrative marked by a continuous transition from fundamental concepts to transformative applications. Initially, the focus was on using the natural biocompatibility of polymers for basic encapsulation. However, this field has matured into the sophisticated engineering of intelligent, multifunctional platforms. Convergence with nanotechnology, advanced materials science, and molecular biology has enabled the design of modern biopolymer systems that can navigate complex biological environments, overcome significant physiological barriers, and deliver therapeutic payloads with unparalleled precision. This evolution has shifted the paradigm from improving drug solubility and stability to enabling targeted, personalized, and combination therapies. We are pleased to present this three-volume series, Biopolymers in Drug and Gene Delivery Systems, which takes readers on an exciting journey from foundational principles to current cutting-edge applications.

The first volume of the Special Issue provided an overview of biopolymer applications in drug delivery (Available online: https://www.mdpi.com/journal/ijms/special_issues/Biopolymers_Drug_Gene_Delivery_Systems (accessed on 15 September 2025)). It emphasized the use of natural polymers, such as cellulose and cyclodextrin, to create advanced gels for controlled release and tissue engineering. Researchers also examined novel systems for delivering challenging therapeutics, such as toxic antibiotics, via polyelectrolyte complexes and hybrid nanoparticles. The volume showcased various biopolymer-based formulations, including films, dressings, and implants designed for localized, targeted administration in fields ranging from ophthalmology to gene therapy.

The second volume of the Special Issue examined cutting-edge trends in biopolymer-based delivery systems (Available online: https://www.mdpi.com/journal/ijms/special_issues/Biopolymers_Delivery_Systems2 (accessed on 15 September 2025)). The Special Issue focused on developing targeted and personalized medicine platforms to improve drug stability and bioavailability. A significant theme was the integration of smart technologies, such as stimuli-responsive polymers, nanotechnology, 3D printing, and electrospinning, to achieve precise control over drug release. The volume also presented innovative methods for noninvasive sustained delivery and multifunctional system design for combination therapies. These methods demonstrate the field’s advancement toward more sophisticated, controllable, and effective therapeutic applications.

This third volume, Biopolymers in Drug and Gene Delivery Systems 3.0 (Available online: https://www.mdpi.com/journal/ijms/special_issues/BIFSHDN056 (accessed on 15 September 2025)), captures the next significant phase in the development of these systems—their maturation toward greater complexity, intelligence, and clinical relevance. The research presented here reflects a field that is solving the intricate puzzles of in vivo efficacy, biological stability, and multimodal treatment, rather than merely proving concepts. Recent comprehensive reviews highlight current trends, such as the push toward theranostics, overcoming biological barriers, and advanced targeting strategies.

Advanced Theranostic Platforms: Integrating therapy and diagnostics into a single platform is a paradigm shift in treatment that moves us toward real-time monitoring and personalization. As reviewed in [1,2], smart polymeric nanocarriers are at the forefront of this revolution. In line with this trend, the research in this manuscript (Contribution 1) explores multifunctional micelles that deliver chemotherapeutic payloads with high efficacy and low toxicity, as well as built-in aggregation-induced emission properties. These systems can provide visual feedback, such as a color change in fluorescence, in response to an acidic tumor microenvironment. This enables drug tracking and could potentially serve as a diagnostic tool, aligning with the broader trend of nanomedicine in cancer theranostics [3].

Conquering Biological Barriers: One of the most significant challenges for gene and oligonucleotide therapies is maintaining stability in the harsh in vivo environment, especially against nucleases and serum proteins. Refs. [4,5] discuss the importance of achieving serum stability for clinical translation. This volume features innovative work (Contribution 2) on engineering robust, serum-resistant ternary polyplexes. These advanced carriers use cross-linked polyanionic coatings and amino acid modifications to effectively shield genetic material, a strategy aligned with the principles of nanoparticle design for overcoming biological barriers [6]. They ensure the safe delivery of genetic material to target cells, significantly enhancing transfection efficiency in vitro and in vivo for applications such as suicide gene therapy.

Expanding the Nucleic Acid Toolkit: The scope of nucleic acid therapeutics is expanding beyond DNA, and siRNA therapy has emerged as a powerful alternative for silencing disease-causing genes [6]. This Special Issue features research (Contribution 3) on anti-angiogenic therapy for complex gynecological conditions, which exemplifies this expansion. Using targeted, peptide-based polymers, researchers have demonstrated highly effective siRNA delivery to silence vascular endothelial growth factor (VEGF), a key driver of pathological blood vessel formation. This approach highlights the potential of biopolymers to enable effective nonsurgical treatment strategies.

Sophisticated Targeting and Localized Release: Maximizing efficacy and minimizing systemic side effects through localized delivery remains a core objective. Advances in materials science are yielding increasingly precise systems [7]. This volume includes research on interpolyelectrolyte complexes (Contribution 4) designed for colon-specific drug delivery. By carefully characterizing the interactions between natural pectins and synthetic polymers, these systems can be adjusted to respond to the colon’s specific pH and enzymatic environment, ensuring targeted release right at the site of action, overcoming biological barriers through advanced material design [8].

Dual-Targeting Strategies for Enhanced Bioavailability: One important application is improving the delivery of difficult-to-deliver drugs, such as potent antibiotics with significant toxicity [9]. In this Special Issue, one manuscript (Contribution 5) describes modifying antibiotic–polysaccharide conjugates with vitamin B12, a strategy that echoes recent advances in polymer modification [10]. This creates a dual-targeting system in which the polymer conjugate provides controlled release and reduced toxicity, while the vitamin B12 moiety facilitates absorption through the gut. This dramatically improves the oral bioavailability of drugs that typically have very poor permeability, addressing challenges similar to those faced in polymyxin delivery systems [11].

In conclusion, this third volume showcases a field that has successfully transitioned from advanced design to overcoming the practical challenges of clinical translation. The focus is on creating intelligent, multifunctional systems that can navigate the complexities of the human body [12] to deliver a diverse range of therapeutics with precision and efficiency. Several key endeavors will likely dominate the future trajectory of biopolymer research. These include the rigorous in vivo validation of sophisticated systems in complex disease models; the development of scalable, reproducible production processes that comply with Good Manufacturing Practices; and the exploration of novel biopolymer sources and hybrid synthetic–biopolymeric materials with enhanced functionality. Proactive engagement with regulatory science is essential for establishing clear approval pathways for these complex products and ensuring the successful clinical adoption of these technologies, particularly in light of new insights into nanoparticle entry into solid tumors [13]. These innovative platforms continue to evolve, showing great potential to transform treatment paradigms. Supported by strong foundational research, as highlighted in previous volumes, they are also endorsed by the broader scientific community. The path forward is clear: translate these remarkable laboratory achievements into safe and effective personalized medicines.

## Data Availability

No new data were generated in this article.
